# ITS-Supported Species Discrimination and ISSR-Based Genetic Diversity and Population Differentiation of *Lumnitzera littorea* in Southern Vietnam

**DOI:** 10.3390/plants15101569

**Published:** 2026-05-21

**Authors:** Duc-Hoan Huynh, The-Kiet Bui-Nguyen, Huu-Nghia Nguyen, Thanh-Cong Nguyen, Hoang-Dung Tran

**Affiliations:** 1 Management Board of Protection and Special-Use Forests of Ho Chi Minh City, 176 Hai Ba Trung Street, Tan Dinh Ward, Ho Chi Minh City 71008, Vietnam; Joankietthe@gmail.com; 2 Faculty of Biotechnology, University of Science, Vietnam National University, Ho Chi Minh City, 227 Nguyen Van Cu Street, Cho Quan Ward, Ho Chi Minh City 72722, Vietnam; rimuruchan1708@gmail.com; 3 Institute of Applied Research and Technology Transfer, Ho Chi Minh City University of Industry and Trade (HUIT), 93 Tan Ky Tan Quy Street, Tan Son Nhi Ward, Ho Chi Minh City 72008, Vietnam; nguyencong0404@gmail.com; 4 Faculty of Biology and Environment, Ho Chi Minh City University of Industry and Trade (HUIT), 140 Le Trong Tan Street, Tay Thanh Ward, Ho Chi Minh City 72009, Vietnam; dungth@huit.edu.vn

**Keywords:** Can Gio, genetic diversity, ISSR, ITS, *Lumnitzera littorea*, population differentiation

## Abstract

*Lumnitzera littorea* is a rare, conservation-relevant mangrove tree with discontinuous records in southern Vietnam, but population-level genetic evidence remains limited. This study combined nuclear rDNA ITS and ISSR markers to distinguish species-level support from population-level comparisons. Can Gio was treated as the focal population, while Dong Nai, Phu Quoc, and Con Dao were used as comparison populations. The 16 study-generated ITS sequences, deposited as PZ348213–PZ348228, supported species-level separation between *L. littorea* and *L. racemosa*, with a between-species p-distance of 0.0459 and 32 fixed diagnostic sites across the 697 bp core. The ISSR matrix comprised 115 individuals and 81 loci, and population analyses were restricted to 110 *L. littorea* individuals. Within this dataset, Dong Nai and Can Gio showed higher ISSR diversity than Con Dao and Phu Quoc. AMOVA indicated significant differentiation among populations (Phi_PT = 0.255, *p* = 0.001), with 74.5% of variation retained within populations; a mainland–island grouping was also significant (Phi_PT = 0.242, *p* = 0.001). Repeated *n* = 20 subsampling retained the relative diversity pattern. The results provide a regional ISSR baseline for conservation-genetics interpretation and support broad representation of local source trees when collecting conservation material; however, they do not define formal management units, official seed-transfer zones, or deep phylogeographic history.

## 1. Introduction

*Lumnitzera* is a non-viviparous mangrove genus in the family Combretaceae and is represented mainly by two closely related species, *L. littorea* and *L. racemosa*, across the Indo-West Pacific [1]. The two species are often discussed together because their ranges partly overlap, but they differ in flower colour, habitat preference, and conservation context. In southern Vietnam, *L. littorea* has been recorded discontinuously from mainland and island mangrove settings, including Can Gio, Dong Nai, Phu Quoc, and Con Dao [2,3,4,5]. The species is conservation-relevant in Vietnam because its populations are small and fragmented, and previous national studies have emphasized morphology, anatomy, associated vegetation, soil conditions, embryo culture, and restoration potential [2,3,4,5,6]. However, these ecological and propagation studies have not yet provided a molecular baseline for comparing Can Gio with nearby mainland and island reference populations.

Southern Vietnam is an informative setting for such a baseline because the sampled localities do not form a single continuous mangrove block. Can Gio and Dong Nai belong to the southeastern mainland coastal-estuarine frame, whereas Phu Quoc and Con Dao are island systems separated from the mainland by marine gaps and different coastal histories. This configuration permits a focused assessment of whether a rare mangrove tree shows only local variation or a detectable mainland–island contrast within a restricted regional dataset. At a broader scale, Su et al. (2007) and Guo et al. (2021) showed that *Lumnitzera* species can display substantial genetic differentiation across the Indo-West Pacific, but those regional studies cannot replace a local southern Vietnam baseline [7,8]. The conceptual gap addressed here is therefore not the absence of data from a single locality, but the lack of a structured genetic baseline for a fragmented mangrove system in which Can Gio, a focal restoration and conservation area, is interpreted against both mainland and island reference populations.

The marker design separated two analytical levels. Nuclear rDNA ITS was used only to support species-level discrimination between *L. littorea* and *L. racemosa* because ITS is widely used for plant barcoding and has shown useful discriminatory power in mangroves and within *Lumnitzera* [9,10,11]. ISSR was used for population-level comparison because it can survey multilocus band polymorphism in a non-model species before codominant or genomic marker sets are standardized [12,13,14]. However, ISSR is a dominant marker system: band presence cannot distinguish heterozygotes from dominant homozygotes, so indices such as He and I must be interpreted as band-based diversity summaries rather than allele-resolved estimates of true heterozygosity or gene flow.

Against this background, this study treats Can Gio as the focal population and uses Dong Nai, Phu Quoc, and Con Dao as comparison populations. The objectives were threefold: first, to support species-level discrimination between *L. littorea* and *L. racemosa* using ITS; second, to describe relative ISSR diversity among four *L. littorea* populations; and third, to assess preliminary among-population differentiation within a conservation-oriented baseline. The study does not aim to reconstruct deep phylogeographic history or define formal management units. Its contribution is a reproducible southern Vietnam baseline that links species validation, dominant-marker population comparison, and cautious conservation interpretation within the same analytical frame.

Because Can Gio was the focal population, the four populations were not treated as symmetrical management units. Dong Nai, Phu Quoc, and Con Dao were included to place Can Gio within the broader variation and differentiation observed in southern Vietnam. Accordingly, the key comparison is how Can Gio relates to a nearby mainland population and two island populations, not whether the present dataset can prescribe source transfer among all localities.

## 2. Results

### 2.1. ITS Supports Species-Level Discrimination Between Lumnitzera littorea and L. racemosa

Within the 16 study-generated specimens, the 14 *L. littorea* samples shared one ITS haplotype, whereas the two *L. racemosa* samples were also mutually consistent. These sequences have been deposited in GenBank as CG1–CG3 = PZ348213–PZ348215; DN1–DN3 = PZ348216–PZ348218; PQ1–PQ4 = PZ348219–PZ348222; CD1–CD4 = PZ348223–PZ348226; and CT1–CT2 = PZ348227–PZ348228. The between-species p-distance within this study-generated set was 0.0459, with 32 fixed diagnostic sites across the 697 bp core. In the main 53-sequence dataset, the maximum within-species distance remained lower than the minimum between-species distance, indicating that the ITS region used here provides species-level barcode support for discriminating *L. littorea* from *L. racemosa* in this material. ITS was therefore retained strictly as a species-support layer, not as evidence for within-species population structure, see Table 1.

**Table 1:** Summary of ITS signal used for species-support interpretation.

Dataset	n *L. littorea*	n *L. racemosa*	Analytical Length	No. of Haplotypes	Within *L. littorea*	Within *L. racemosa*	Between Species	Notes
16-sample study set	14	2	697 bp (shared core)	*L. littorea* = 1; *L. racemosa* = 1	0.000	0.000	0.0459	32 fixed diagnostic sites between species
Main 53-sequence set	26	27	714 columns; 697 bp gap-free core	*L. littorea* = 5; *L. racemosa* = 4	0.000–0.0086	0.000–0.0228	0.0459–0.0545	Barcode gap retained

Note: Between-species values refer to the *L. littorea* versus *L. racemosa* comparison for the stated ITS dataset; this table supports species-level interpretation only.

**Figure 1:**
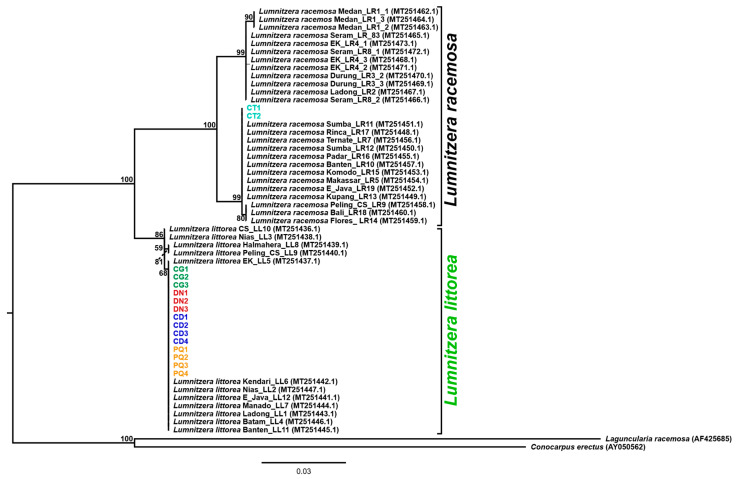
Rooted ITS phylogeny used for species-level support between *Lumnitzera littorea* and *L. racemosa*. The tree was inferred from 55 sequences and rooted with *Laguncularia racemosa* AF425685 and *Conocarpus erectus* AY050562. Study-generated *L. littorea* samples are shown with colored terminal labels and GenBank accession numbers: CG1–CG3, Can Gio, green, PZ348213–PZ348215; DN1–DN3, Dong Nai, red, PZ348216–PZ348218; CD1–CD4, Con Dao, blue, PZ348223–PZ348226; and PQ1–PQ4, Phu Quoc, orange, PZ348219–PZ348222. Study-generated *L. racemosa* samples are shown in cyan: CT1–CT2, PZ348227–PZ348228. Support values are shown at internal nodes. The figure supports species-level separation only and is not used for within-species population-structure inference.

The rooted ITS tree recovered *L. littorea* and *L. racemosa* as distinct species-level groups (Figure 1), consistent with the barcode-gap pattern and the fixed diagnostic sites observed in the present dataset. Support values are shown at the major internal nodes of the tree. The rooted topology therefore supports species-level separation between *L. littorea* and *L. racemosa* but does not justify within-species population-structure inference.

### 2.2. ISSR Marker Properties in 110 L. littorea Individuals

The 12 ISSR primers generated 81 loci in the 110-individual analytical set of *L. littorea* (Table 2). All 81 loci were polymorphic across the full analytical set, indicating that the marker panel provided sufficient information for a baseline population-level comparison. Mean He by primer ranged from 0.150 for primer 876 to 0.495 for primer 842, and mean I ranged from 0.283 to 0.688. Because these values are derived from dominant ISSR bands, they are used here as relative marker summaries rather than allele-resolved heterozygosity estimates. In general, primers 842 and 868 were relatively more informative, whereas primer 876 contributed only one locus and should therefore be regarded as supportive rather than central to interpretation.

### 2.3. ISSR Diversity at Population Level

ISSR indices showed that genetic variation was not evenly distributed among the four surveyed populations (Table 3). Dong Nai (He = 0.357; I = 0.517) and Can Gio (He = 0.351; I = 0.512) had relatively higher diversity in the current dataset, followed by Con Dao (He = 0.293; I = 0.435), whereas Phu Quoc was lower (He = 0.224; I = 0.346). Across all 110 *L. littorea* individuals, all 81 loci were polymorphic, with He = 0.398 and I = 0.579. Interpreted as a relative pattern rather than an exact numerical equivalence, Dong Nai and Can Gio formed the higher-diversity group, Con Dao was intermediate, and Phu Quoc was lower.

Because sample sizes were unequal, a sensitivity check was performed by randomly subsampling Can Gio, Dong Nai, and Phu Quoc to *n* = 20 without replacement for 1000 iterations to match the Con Dao sample size. A fixed random seed (10 May 2026) was used for reproducibility. The resampled mean He values were 0.345 for Can Gio, 0.350 for Dong Nai, and 0.221 for Phu Quoc; the corresponding 95% resampling intervals were 0.326–0.356, 0.299–0.375, and 0.213–0.228. These results retained the same broad interpretation as the full dataset: Can Gio and Dong Nai remained higher than Phu Quoc, while Con Dao retained an intermediate observed value (He = 0.293). Thus, the lower Phu Quoc diversity signal and the relatively high Can Gio/Dong Nai signal were not artifacts of comparing *n* = 30 populations with the *n* = 20 Con Dao sample, although comparisons involving Con Dao should still be interpreted cautiously.

### 2.4. Among-Population Differentiation and Preliminary Spatial Structure

AMOVA indicated significant overall differentiation among the four populations (Phi_PT = 0.255, *p* = 0.001). When the data were grouped into mainland and island populations, the signal remained significant (Phi_PT = 0.242, *p* = 0.001). The four-population AMOVA also showed that 74.5% of the variation resided within populations, while 25.5% was partitioned among populations (Table 4). Thus, most variation resided within populations, but the among-population component was large enough to support cautious discussion of population differentiation within the current dataset. This result does not by itself justify deep phylogeographic reconstruction.

**Figure 2:**
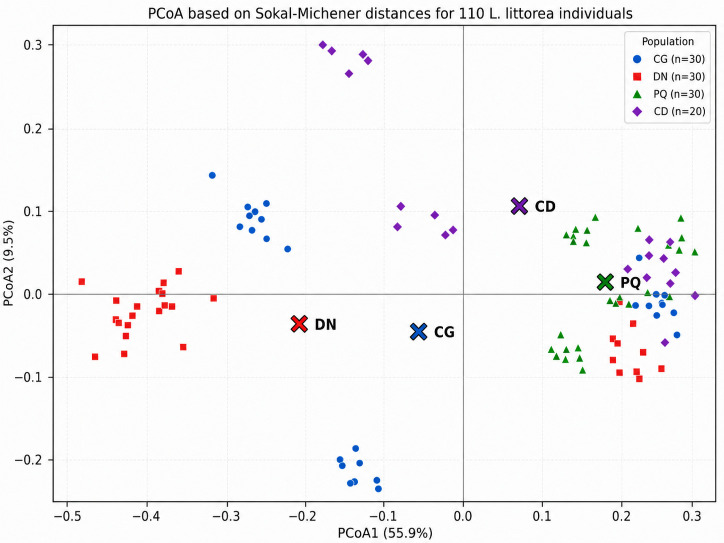
Principal coordinates analysis (PCoA) of 110 *L. littorea* individuals, visualizing Sokal–Michener dissimilarities calculated from the dominant ISSR band matrix. Axis 1 explains 55.9% and axis 2 explains 9.5% of the variance. Colored symbols represent individual samples, and larger X symbols indicate population centroids for CG, DN, PQ, and CD. The ordination provides a descriptive visualization of population-level separation, with exact pairwise Phi_PT values reported in Table 5; it is not interpreted as a test of gene flow.

At the population-pair level, Can Gio-Dong Nai was the closest pair (Phi_PT = 0.133), whereas Dong Nai-Phu Quoc was the most differentiated (Phi_PT = 0.407; Table 5). All remaining pairs also differed significantly at *p* ≤ 0.003. Table 5 is retained in the main text because it reports the exact pairwise Phi_PT values and permutation *p* values; the heatmap has been moved to Supplementary Figure S1 to avoid redundancy while preserving a visual summary for readers. The PCoA based on the same Sokal–Michener dissimilarity matrix provides a complementary ordination view: the population centroids do not form a single cluster, Dong Nai and Phu Quoc occupy separated positions along the main ordination axis, and Con Dao is displaced mainly along the second axis (Figure 2). This supports a descriptive interpretation of population differentiation and relative spatial separation, not a stronger historical gene-flow claim.

## 3. Discussion

### 3.1. ITS Is a Species-Support Layer, Not a Population Marker

The ITS results support a species-validation role rather than a population-marker role. The 16 study-generated specimens showed a uniform ITS signal within the 14 *L. littorea* samples and a clear separation from the two *L. racemosa* samples, while the main 53-sequence dataset retained a gap between within-species and between-species distances. This reading is in line with Nguyen et al. (2017), who found that ITS provided high discriminatory resolution in mangroves and was more informative than the rbcL/matK combination within *Lumnitzera* [9]. At the same time, ITS has limited within-species resolution for the present question and cannot replace the ISSR population-level dataset. Natural hybridization has also been reported within *Lumnitzera* [15], further supporting an ITS interpretation limited to species validation. In this study, ITS should therefore be regarded as species-level validation for the present sample set, not as a basis for broader population-genetic conclusions.

### 3.2. ISSR Diversity and Structure in the Southern Vietnam Baseline

At the ISSR level, the primary pattern was that Dong Nai and Can Gio formed the relatively higher-diversity group, Con Dao was intermediate, and Phu Quoc was lower. This pattern should be interpreted within this study because ISSR band frequencies and derived indices are sensitive to primer set, scoring rules, and sampling design. For that reason, the present analysis does not rely on direct comparison of absolute He values with other ISSR studies. Instead, it asks how the four southern Vietnamese populations are ordered within the same 81-locus matrix. The AMOVA result adds a second point: approximately three-quarters of the variation was within populations, but among-population differentiation was still significant. This combination is biologically plausible for a rare, fragmented mangrove species: populations retain internal band variation, yet the regional dataset still carries a detectable spatial signal. Guo et al. (2021), using chloroplast DNA and nuclear microsatellites across the Indo-West Pacific, addressed phylogeographic and genetic differentiation patterns in both *L. littorea* and *L. racemosa*, although the marker systems and geographic scale differ from the present ISSR baseline [8].

### 3.3. Plausible Drivers of the Observed Population Pattern

The observed pattern is compatible with several non-exclusive explanations, but the present dataset cannot distinguish among them definitively. Can Gio and Dong Nai are both mainland southeastern localities and form the closest pair in the pairwise Phi_PT table. The Phu Quoc population is a southwestern island population and shows the lowest ISSR diversity indices as well as the strongest differentiation from Dong Nai. This pattern could reflect island isolation, founder effects, genetic drift in a smaller or more discontinuous local population, limits to hydrochorous propagule exchange, or coastal discontinuity between the southeastern mainland and the southwestern island setting. Con Dao is also insular but occupies an intermediate position in the diversity table and ordination, indicating that island status alone is not a sufficient explanation. Vietnamese ecological studies further show that localities supporting *L. littorea* differ in leaf anatomical traits, associated vegetation, and habitat context [2,4,5]. The safest interpretation is therefore that the ISSR data detect population differentiation and a mainland–island signal, while the underlying causes remain hypotheses for future studies with balanced sampling and codominant or genomic markers.

In the broader mangrove-genetics literature, the southern Vietnam pattern is consistent with the general expectation that population structure can emerge when dispersal corridors are interrupted by land barriers, oceanographic discontinuities, or asymmetric propagule exchange. Reviews and comparative studies have emphasized that mangrove gene-flow patterns depend on marker type, dispersal mode, and coastal configuration; examples from *Rhizophora apiculata*, *Rhizophora mucronata*, and *Rhizophora stylosa* across the Indo-West Pacific, *Ceriops tagal* in Southeast Asia, and *Avicennia marina* in the Malay Peninsula show that coastal and land-barrier settings can structure mangrove populations even when taxa occupy broad coastal ranges [16,17,18,19]. These studies are used here as contextual analogues, not as numerical comparators to the ISSR He values reported for *L. littorea*.

### 3.4. Conservation Implications and Marker Limitations

From an applied perspective, the dataset helps avoid two opposite errors: treating all localities as genetically interchangeable or assigning rigid management units before the evidence is strong enough. Can Gio and Dong Nai retained relatively high ISSR diversity within the current comparison frame, whereas Phu Quoc was lower and Dong Nai-Phu Quoc was the most differentiated pair. For conservation and restoration planning, this supports broad local source-tree representation in Can Gio and other source areas, rather than collection from a small number of convenient individuals. It also supports caution when considering material exchange between highly differentiated provenances, particularly between Dong Nai and Phu Quoc, until stronger evidence is available. Because neither marker layer resolves parentage or seed-source lineages, the conservation implication is restricted to broad local source-tree representation. The results should also be interpreted within the limits of a dominant-marker dataset. ISSR-derived PPB, Na, Ne, He, I, PIC, and Phi_PT are band-based comparative summaries, not direct estimates of allele frequencies, true heterozygosity, migration rate, or demographic history. The present dataset can inform cautious source representation and the design of follow-up sampling, but it does not establish formal management units, official seed-transfer zones, prescriptive mixing schemes, or deep phylogeographic history.

## 4. Materials and Methods

### 4.1. Sampling Framework and Analytical Scope

**Figure 3:**
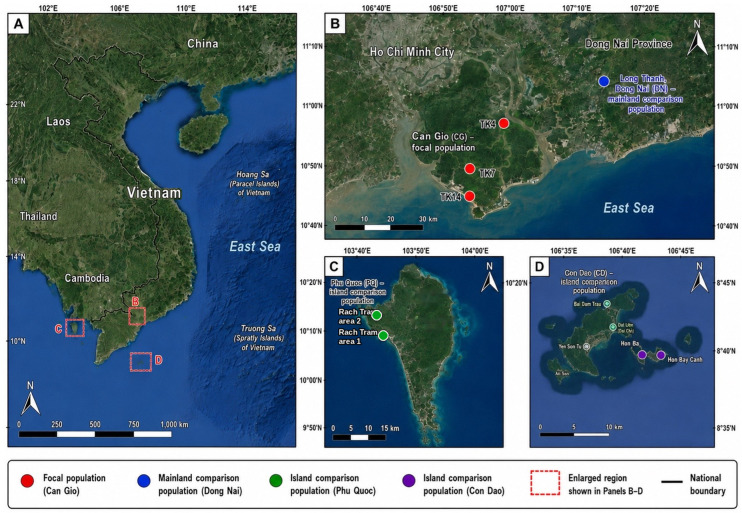
Sampling localities used in the Can Gio-focused comparison framework. (**A**) Full-country Vietnam locator map showing the enlarged regions presented in panels (**B**–**D**). (**B**) Can Gio (CG) focal population and the Dong Nai (DN) mainland comparison population. Red points indicate the Can Gio sampling sites TK4, TK7, and TK14; the blue point indicates the Long Thanh, Dong Nai comparison population. (**C**) Phu Quoc (PQ) island comparison population, showing the Rach Tram area 1 and Rach Tram area 2 sampling sites. (**D**) Con Dao (CD) island comparison population, showing the Hon Ba and Hon Bay Canh sampling sites. Dashed red rectangles in panel A indicate the enlarged regions shown in panels (**B**–**D**). Colored points correspond to population groups as indicated in the legend.

Can Gio was treated as the focal population, whereas Dong Nai, Phu Quoc, and Con Dao served as comparison populations. Two molecular data layers with different analytical scopes were used. The ITS layer comprised 16 barcode specimens, including 14 *L. littorea* and 2 *L. racemosa*, and was used only to support species identification. The study-generated ITS specimens have the following GenBank accessions: CG1–CG3 = PZ348213–PZ348215; DN1–DN3 = PZ348216–PZ348218; PQ1–PQ4 = PZ348219–PZ348222; CD1–CD4 = PZ348223–PZ348226; CT1–CT2 = PZ348227–PZ348228. The ISSR layer consisted of the final binary matrix of 115 samples × 81 loci, including 110 *L. littorea* individuals [30 from Can Gio (CG), 30 from Dong Nai (DN), 30 from Phu Quoc (PQ), and 20 from Con Dao (CD)] plus 5 *L. racemosa* samples (CT1–CT5). All diversity and population-structure analyses were restricted to the 110 *L. littorea* individuals; the CT samples were retained only as interspecific references when needed for visualization. The relative positions of the sampled localities are shown in Figure 3, which identifies the Can Gio sublocalities, the Phu Quoc and Con Dao island sites, and the Dong Nai locality used in the Can Gio-focused comparison.

### 4.2. DNA Extraction, PCR, and Electrophoresis

Total DNA was extracted from leaf tissue using a modified CTAB protocol. Briefly, approximately 100 mg of dried leaf tissue was ground in liquid nitrogen and mixed with 700 µL of preheated 2% CTAB buffer (100 mM Tris-HCl, pH 8.0; 20 mM EDTA; 1.4 M NaCl; 1% PVP-40; and 0.2% beta-mercaptoethanol). The mixture was incubated at 65 °C for 30 min, extracted with chloroform:isoamyl alcohol (24:1), precipitated with cold isopropanol, washed with 70% ethanol, and dissolved in 50 µL TE buffer. DNA quality was checked on 0.8% agarose gels in 1× TBE, stained with SYBR Safe 1× for 10–15 min, and visualized on a 470 nm blue LED transilluminator. The ITS region of nuclear rDNA was amplified with ITS5A (5′-CCTTATCATTTAGAGGAAGGAG-3′) and ITS4 (5′-TCCTCCGCTTATTGATATGC-3′). Each 25 µL ITS PCR contained approximately 50 ng template DNA, 2.5 µL 10× PCR buffer containing 1.5 mM MgCl2, 0.2 mM each dNTP, 0.4 µM each primer, 1 U Taq DNA polymerase, and nuclease-free water to final volume. The ITS cycling profile was 95 °C for 4 min; 35 cycles of 95 °C for 40 s, 52 °C for 30 s, and 72 °C for 35 s; and a final extension at 72 °C for 5 min. ITS amplicons were checked on 1.5% agarose gels in 1× TBE with a 1 kb ladder, purified using the GeneJET PCR Purification Kit (Thermo Fisher Scientific, Waltham, MA, USA), and Sanger-sequenced bidirectionally on an ABI PRISM 3730xl platform (Applied Biosystems, Foster City, CA, USA). For ISSR, 12 primers (808, 823, 836, 840, 841, 842, 844, 845, 868, 873, 874, and 876) were used to generate DNA fingerprints across the full sample system. These primers correspond to a subset of the UBC primer set no. 9, follow the panel previously applied to *L. littorea* by Su et al. (2007) [7], and are listed with sequence information in Supplementary Table S3. ISSR amplification used the same 25 µL reaction system, except that the corresponding single ISSR primer was used at 0.4 µM. The ISSR cycling profile was 94 °C for 5 min; 40 cycles of 94 °C for 1 min, 52 °C for 65 s, and 72 °C for 2 min; and a final extension at 72 °C for 10 min. ISSR products were separated on 2.0% agarose gels in 1× TBE with a 1 kb ladder. Gels were stained with SYBR Safe 1× for 10–15 min, visualized on a 470 nm blue LED transilluminator, and photographed digitally. Only bands that were clear, sharp, and positionally stable were retained for binary scoring.

### 4.3. ITS Data Processing

The ITS data were used exclusively to support species-level discrimination. The formal ITS dataset comprised 53 *Lumnitzera* sequences, including 16 newly sequenced specimens from this study (14 *L. littorea* and 2 *L. racemosa*) and reference sequences retrieved from GenBank. The study-generated sequence headers and manifest use sample|accession notation for CG1–CG3 = PZ348213–PZ348215; DN1–DN3 = PZ348216–PZ348218; PQ1–PQ4 = PZ348219–PZ348222; CD1–CD4 = PZ348223–PZ348226; and CT1–CT2 = PZ348227–PZ348228. The main 53-sequence ingroup alignment contained 714 columns. The 697 bp shared gap-free core used for distance summaries was generated by deleting columns containing gaps in any of the 53 aligned sequences rather than by individually stripping gaps from each sequence. Two outgroup sequences (*Laguncularia racemosa* AF425685 and *Conocarpus erectus* AY050562) were prepared separately for rooted-tree visualization but were not included in the formal 53-sequence summary statistics. ITS results were interpreted only as support for the species boundary between *L. littorea* and *L. racemosa*, not as evidence for within-species population structure in *L. littorea*.

For the rooted ITS phylogeny used as species-support evidence, the 53-sequence *Lumnitzera* dataset was combined with two outgroups and analyzed in IQ-TREE 3.0.1 after alignment and end trimming of the ITS region. The main *Lumnitzera* alignment used for species-level summaries contained 714 columns, and its shared gap-free core was 697 bp. In the rooted-tree sequence set, the *Lumnitzera* records retained 697–701 bp after trimming, whereas the two outgroups retained 614 bp and 601 bp; the tree output and sequence manifest are provided in Supplementary Dataset S1. ModelFinder selected HKY+F+I by BIC, and *Laguncularia racemosa* AF425685 and *Conocarpus erectus* AY050562 were used as outgroups. This rooted tree was interpreted only as support for species-level separation between *L. littorea* and *L. racemosa* and not as evidence for within-species population structure.

### 4.4. ISSR Data Processing and Statistical Analysis

ISSR bands were scored from gel images as a binary matrix using the convention 1 = band present and 0 = band absent after standardized lane-by-lane reading across gels. Only bands that were clear, sharp, positionally stable, and consistently readable were retained; faint, smeared, or ambiguous bands were excluded from the matrix. The full matrix contained 115 individuals × 81 loci, but all population-level summaries were calculated only for the 110 *L. littorea* individuals; CT1-CT5 of *L. racemosa* were retained only as interspecific references when needed for visualization. The final binary matrix was curated in Microsoft Excel and analyzed in GenAlEx 6.5 [20] for PPB, Na, Ne, He, I, AMOVA, pairwise Phi_PT, and principal coordinates analysis. The PCoA shown in Figure 2 was derived from the Sokal–Michener dissimilarity matrix calculated for the same 110-individual analytical set. AMOVA and pairwise Phi_PT significance were evaluated with 999 permutations. Because ISSR is a dominant marker system, band presence cannot distinguish heterozygotes from dominant homozygotes. Therefore, PPB, Na, Ne, He, I, PIC, and Phi_PT were treated as band-based comparative summaries rather than direct estimates of allele frequency, true heterozygosity, gene flow, or demographic history. To examine sensitivity to the smaller Con Dao sample size, Can Gio, Dong Nai, and Phu Quoc were randomly subsampled to *n* = 20 without replacement for 1000 iterations each using Python 3 with a fixed random seed of 20260510. For each iteration, PPB, Na, Ne, He, and I were recalculated from the 81-locus binary matrix, and the resampled means and 2.5–97.5 percentile intervals were compared with the observed Con Dao values. The resampling script is provided as Supplementary Code S1.

### 4.5. Data Provenance and Supplementary Materials

To avoid duplication between the main text and the Supplementary Materials, this section is limited to the role of each supporting file. Supplementary Table S1 summarizes the analytical layers and sample roles, Supplementary Table S2 summarizes the four *L. littorea* populations used in the population-level analyses, and Supplementary Table S3 reports the confirmed ISSR motif notation and expanded 5′-3′ primer sequence information. Supplementary Dataset S1 provides the 16 study-generated ITS sequences with GenBank accessions, the main 53-sequence alignment, the 697 bp shared gap-free core alignment, the rooted-tree input sequence set and tree output, and the ITS sequence manifest. Supplementary Dataset S2 provides the final ISSR binary matrix, Supplementary Dataset S3 provides the sample metadata and dataset-role fields, Supplementary Code S1 provides the *n* = 20 resampling script, Supplementary Dataset S4 provides the resampling summary, and Supplementary Figure S1 provides the pairwise Phi_PT heatmap as a supplementary visualization of the exact pairwise values reported in Table 5.

## 5. Conclusions

This study provides a genetic baseline for *L. littorea* in southern Vietnam by combining ITS species-support evidence with ISSR-based comparative population summaries and using Can Gio as the focal population. ITS supports discrimination between *L. littorea* and *L. racemosa* in the present material, whereas ISSR places Can Gio and Dong Nai among the relatively higher-diversity populations in the current comparison frame and shows significant among-population differentiation at the level of band data. The *n* = 20 resampling sensitivity check indicates that the main diversity pattern is not an artifact of comparing three *n* = 30 populations with Con Dao at *n* = 20. The practical implication is that conservation and restoration work in Can Gio should maintain broad local source-tree representation and treat highly differentiated provenances with caution. The study does not define formal management units, official seed-transfer zones, or deep phylogeographic history. Its main contribution is a reproducible regional baseline that can inform near-term conservation choices and support future follow-up with balanced sampling and codominant or higher-density genomic markers.

## Figures and Tables

**Table 2:** ISSR marker properties by primer in 110 *L. littorea* individuals.

Primer	No. of Loci	Polymorphic Loci	PPB (%)	Mean He	Mean I	Mean PIC
808	8	8	100.0	0.366	0.549	0.366
823	9	9	100.0	0.255	0.399	0.255
836	8	8	100.0	0.422	0.611	0.422
840	8	8	100.0	0.433	0.620	0.433
841	7	7	100.0	0.465	0.656	0.465
842	5	5	100.0	0.495	0.688	0.495
844	3	3	100.0	0.464	0.657	0.464
845	6	6	100.0	0.337	0.516	0.337
868	6	6	100.0	0.474	0.667	0.474
873	9	9	100.0	0.345	0.524	0.345
874	11	11	100.0	0.444	0.636	0.444
876	1	1	100.0	0.150	0.283	0.150

Note: PPB = percentage of polymorphic bands; He = Nei’s gene diversity calculated from dominant ISSR band data; I = Shannon’s information index; PIC = polymorphism information content. Values are used as comparative band-based summaries, not allele-resolved heterozygosity estimates.

**Table 3:** ISSR diversity indices by population and for all 110 *L. littorea* individuals.

Population	n	Polymorphic Loci	PPB (%)	Na	Ne	He	I
CG	30	75	92.6	1.926	1.632	0.351	0.512
DN	30	73	90.1	1.901	1.645	0.357	0.517
PQ	30	65	80.2	1.802	1.374	0.224	0.346
CD	20	67	82.7	1.827	1.511	0.293	0.435
All *L. littorea* (*n* = 110)	110	81	100.0	2.000	1.711	0.398	0.579

Note: Population codes are CG = Can Gio, DN = Dong Nai, PQ = Phu Quoc, and CD = Con Dao. PPB = percentage of polymorphic bands; Na = observed number of alleles/band states; Ne = effective number of alleles; He = Nei’s gene diversity; I = Shannon’s information index. For ISSR, these are dominant band-based summaries.

**Table 4:** AMOVA summary under two grouping scenarios.

Scenario	Phi_PT	*p*	Among-Group Variation	Within-Group Variation
Four separate populations	0.255	0.001	25.5%	74.5%
Mainland–island grouping	0.242	0.001	24.2%	75.8%

Note: Phi_PT is the AMOVA analogue of population differentiation used for dominant binary data in GenAlEx; *p* values are based on 999 permutations. Percentages report the variance components assigned to among- and within-group levels in each scenario.

**Table 5:** Pairwise Phi_PT among *L. littorea* populations.

Population 1	Population 2	Phi_PT	Permutation *p*
CG	DN	0.133	0.003
CG	PQ	0.256	0.001
CG	CD	0.172	0.002
DN	PQ	0.407	0.001
DN	CD	0.303	0.001
PQ	CD	0.163	0.001

Note: Phi_PT values are pairwise differentiation estimates from the same 110-individual ISSR band matrix; *p* values are based on 999 permutations. Exact values are retained in the main table, while the heatmap visualization is provided as Supplementary Figure S1.

## Data Availability

The data supporting the findings of this study are available within the article and its Supplementary Materials. The 16 ITS sequences newly generated in this study have been deposited in GenBank under the following accession numbers: CG1, PZ348213; CG2, PZ348214; CG3, PZ348215; DN1, PZ348216; DN2, PZ348217; DN3, PZ348218; PQ1, PZ348219; PQ2, PZ348220; PQ3, PZ348221; PQ4, PZ348222; CD1, PZ348223; CD2, PZ348224; CD3, PZ348225; CD4, PZ348226; CT1, PZ348227; and CT2, PZ348228. Supplementary Dataset S1 provides the ITS sequence files and accession manifest. Supplementary Dataset S2 provides the final ISSR binary matrix (115 individuals × 81 loci), and Supplementary Dataset S3 provides the sample metadata and dataset-role fields used to define the 110-individual *L. littorea* analytical set and the 16 study-generated ITS specimens.

## Supplementary Materials

The following supporting information can be downloaded at: https://www.mdpi.com/article/10.3390/plants15101569/s1, Supplementary Table S1, analytical layers and sample roles used in the study; Supplementary Table S2, population summary and analytical framing for the four *L. littorea* localities; Supplementary Table S3, ISSR primers used in the study, with motif notation, expanded 5′-3′ primer sequence information, IUPAC ambiguity codes, and loci retained; Supplementary Dataset S1, ITS sequences, GenBank accession manifest, main 53-sequence alignment, 697 bp shared gap-free core alignment, rooted-tree input sequence set, and rooted-tree output; Supplementary Dataset S2, ISSR binary matrix (115 individuals × 81 loci); Supplementary Dataset S3, sample metadata and dataset-role fields; Supplementary Code S1, reproducible *n* = 20 resampling sensitivity script; Supplementary Dataset S4, resampling sensitivity summary; Supplementary Figure S1, heatmap visualization of pairwise Phi_PT values.
