# Supplementary material for: ITS-Supported Species Discrimination and ISSR-Based Genetic Diversity and Population Differentiation of Lumnitzera littorea in Southern Vietnam
Source: Plants (Basel). 2026 May 21;15(10):1569. doi: 10.3390/plants15101569 (PMC13210803; doi:10.3390/plants15101569)
Supplement: Supplementary file 1 [file plants-15-01569-s001.zip › plants-4292459_V2_Supplementary_Information.pdf]

Supplementary Information

ITS-supported species discrimination and ISSR-based genetic diversity and population differentiation of *Lumnitzera littorea* in southern Vietnam

This Supplementary Information provides the analytical-layer map, population summary, confirmed ISSR primer information, and supporting-file inventory for the revised manuscript.

S1. Analytical layers and sample roles

Supplementary Table S1. Analytical layers and sample roles used in the study.

Layer / data component	Composition	Analytical role	Included in population statistics?
Study-generated ITS set	16 specimens: 14 *L. littorea* (CG1-CG3, DN1-DN3, PQ1-PQ4, CD1-CD4) and 2 *L. racemosa* (CT1-CT2); GenBank accessions PZ348213-PZ348228	Species-level discrimination support between *L. littorea* and *L. racemosa*	No
Main ITS dataset	53 Lumnitzera sequences; 714 aligned columns; 697 bp shared gap-free core produced by deleting columns containing gaps in any of the 53 Lumnitzera sequences	Barcode-gap and species-support summary	No
Rooted ITS visualization set	55-sequence rooted-tree input set, including *Laguncularia racemosa* AF425685 and *Conocarpus erectus* AY050562; trimmed/gap-stripped lengths are	Rooted phylogeny for species-level visualization	No

	697-701 bp for Lumnitzera records and 601-614 bp for the outgroup records; rooted-tree input sequence set and Newick output are provided in Supplementary Dataset S1		
Full ISSR matrix	115 individuals × 81 loci, including 110 *L. littorea* individuals and 5 *L. racemosa* reference samples (CT1-CT5)	Binary band matrix and interspecific reference visualization when needed	No for CT references; *L. littorea* subset used for statistics
L. littorea ISSR analytical set	110 *L. littorea* individuals: CG = 30, DN = 30, PQ = 30, CD = 20	Population-level diversity, AMOVA, pairwise Phi_PT, and PCoA	Yes
Resampling sensitivity set	1,000 random subsamples without replacement from CG, DN, and PQ to n = 20; random seed = 20260510	Sensitivity check for unequal sampling relative to CD n = 20	Derived check

S2. Population summary and analytical framing

Supplementary Table S2. Population summary and analytical framing for the four *L. littorea* localities.

Abbreviation	Locality	Analytical role	n *L. littorea*	Notes
CG	Can Gio	Focal population	30	Southeastern mainland coastal-estuarine setting; one of the relatively higher-diversity populations in the ISSR matrix.
DN	Dong Nai	Mainland comparison/reference population	30	Nearby mainland comparison/reference locality; closest pair with CG in pairwise Phi_PT.
PQ	Phu Quoc	Island	30	Southwestern island

		comparison/reference population		setting; lower ISSR diversity and strongest pairwise differentiation from DN.
CD	Con Dao	Island comparison/reference population	20	Island setting; smaller sample size, interpreted cautiously; observed diversity is intermediate.

S3. ISSR primers

Supplementary Table S3. ISSR primers used in the study, with motif notation, expanded 5'-3' primer sequence, IUPAC ambiguity codes, and loci retained in the 110-individual *L. littorea* analytical set.

Primer	Motif notation	Expanded primer sequence (5′-3′)	IUPAC note	No. of loci retained
808	(AG)8C	AGAGAGAGAGAGAGAGC	Anchored dinucleotide repeat	8
823	(TC)8C	TCTCTCTCTCTCTCTCC	Anchored dinucleotide repeat	9
836	(AG)8YA	AGAGAGAGAGAGAGAGYA	Y = C or T	8
840	(GA)8YT	GAGAGAGAGAGAGAGAYT	Y = C or T	8
841	(GA)8YC	GAGAGAGAGAGAGAGAYC	Y = C or T	7
842	(GA)8YG	GAGAGAGAGAGAGAGAYG	Y = C or T	5
844	(CT)8RC	CTCTCTCTCTCTCTCTRC	R = A or G	3
845	(CT)8RG	CTCTCTCTCTCTCTCTRG	R = A or G	6
868	(GAA)6	GAAGAAGAAGAAGAAGAA	Tri-nucleotide repeat	6
873	(GACA)4	GACAGACAGACAGACA	Tetra-nucleotide repeat	9
874	(CCCT)4	CCCTCCCTCCCTCCCT	Tetra-nucleotide repeat	11
876	(GATA)2(GACA)2	GATAGATAGACAGACA	Mixed repeat	1

S4. Supplementary files

Supplementary Table S1 file (plants-4292459_V2_Supplementary_Table_S1_analytical_layers_and_sample_roles.csv): CSV version of Supplementary Table S1 for direct inspection or reuse.

Supplementary Table S2 file (plants-4292459_V2_Supplementary_Table_S2_population_summary.csv): CSV version of Supplementary Table S2 for direct inspection or reuse.

Supplementary Table S3 file (plants-4292459_V2_Supplementary_Table_S3_ISSR_primers.csv): CSV version of Supplementary Table S3, including confirmed ISSR motif notation and expanded 5'-3' primer sequence information.
Supplementary Dataset S1 (plants-4292459_V2_Supplementary_Dataset_S1_ITS_sequences_and_manifest.zip): ITS FASTA files, GenBank accession manifest, main 53-sequence alignment, 697 bp shared gap-free core alignment, rooted-tree input sequence set, and rooted-tree Newick output.

Supplementary Dataset S2 (plants-4292459_V2_Supplementary_Dataset_S2_ISSR_binary_matrix.csv): Final ISSR binary matrix. Supplementary Dataset S2 is stored with loci in rows and samples in columns, corresponding to a 115-individual × 81-locus ISSR binary matrix.

Supplementary Dataset S3 (plants-4292459_V2_Supplementary_Dataset_S3_sample_metadata.csv): Sample identities, locality labels, species assignments, and dataset-role fields.

Supplementary Code S1 (plants-4292459_V2_Supplementary_Code_S1_resampling_sensitivity.py): Python script for n = 20 resampling sensitivity analysis with random seed = 20260510.

Supplementary Dataset S4 (plants-4292459_V2_Supplementary_Dataset_S4_resampling_sensitivity_summary_seed20260510.csv): Resampling sensitivity summary table generated from Supplementary Code S1.

Supplementary Figure S1 (plants-4292459_V2_Supplementary_Figure_S1_pairwise_PhiPT_heatmap.png): Pairwise Phi_PT heatmap provided as a supplementary visual summary of Table 5; it was moved from the main text to avoid redundancy with exact pairwise values.

S5. Interpretation notes

ITS is interpreted only as species-level support between *L. littorea* and *L. racemosa*; it is not used as evidence for within-species population structure.

ISSR is a dominant, band-based marker system. PPB, Na, Ne, *He*, I, PIC, Phi_PT, AMOVA, and PCoA are interpreted as comparative band-data summaries, not allele-resolved estimates of true heterozygosity, migration rate, or demographic history.

Con Dao has n = 20, whereas CG, DN, and PQ each have n = 30. The resampling sensitivity check matches CG, DN, and PQ to n = 20 to evaluate whether the main diversity pattern is driven by unequal sampling.
