# Supplementary figures and images for: ITS-Supported Species Discrimination and ISSR-Based Genetic Diversity and Population Differentiation of Lumnitzera littorea in Southern Vietnam
Source: Plants (Basel). 2026 May 21;15(10):1569. doi: 10.3390/plants15101569 (PMC13210803; doi:10.3390/plants15101569)

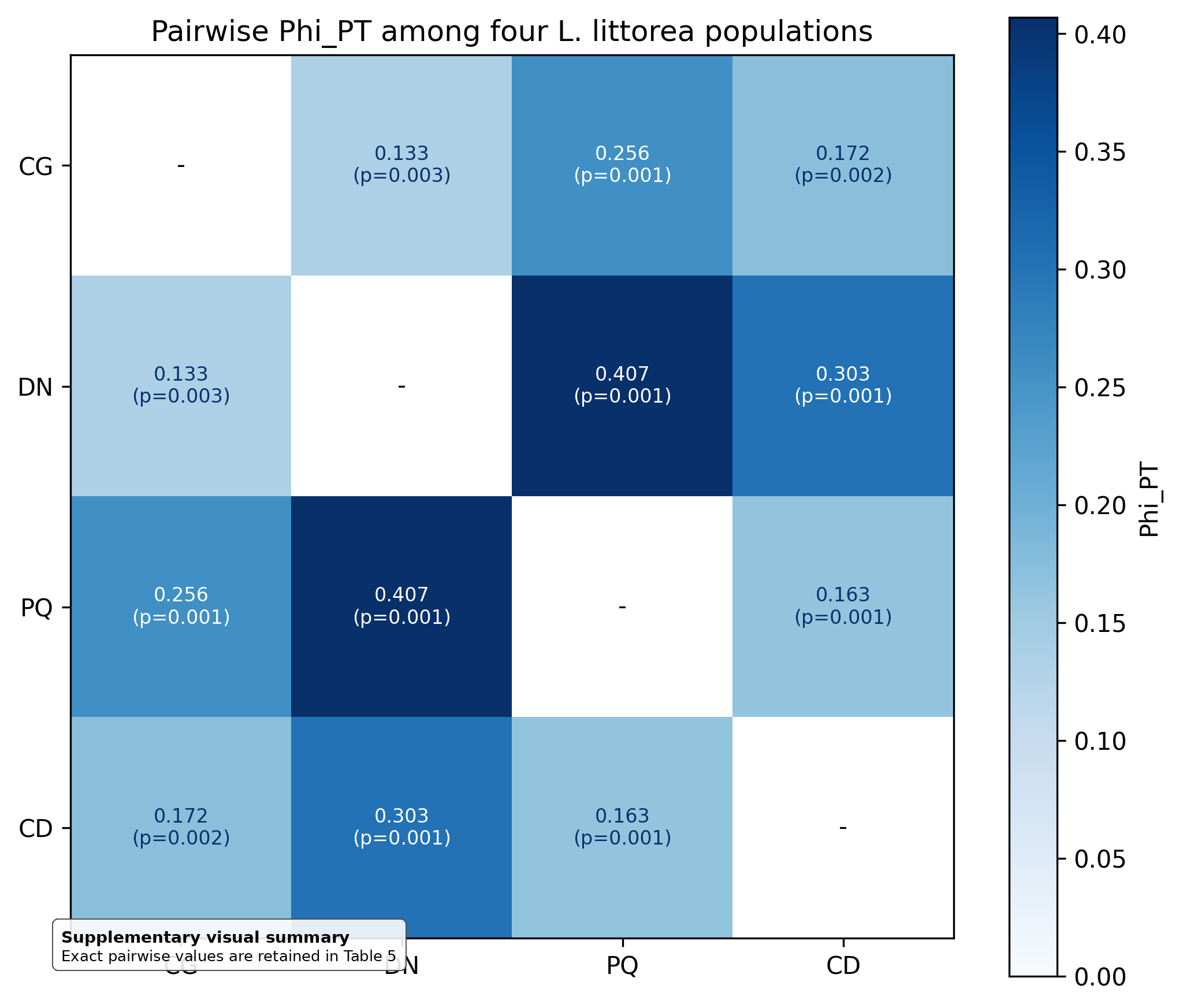

Supplement: Supplementary file 1 [file plants-15-01569-s001.zip › plants-4292459_V2_Supplementary_Figure_S1_pairwise_PhiPT_heatmap.png]
